# Problematic smartphone use is associated with differences in static and dynamic brain functional connectivity in young adults

**DOI:** 10.3389/fnins.2022.1010488

**Published:** 2022-10-21

**Authors:** Dayi Liu, Xiaoxuan Liu, Yicheng Long, Zhibiao Xiang, Zhipeng Wu, Zhening Liu, Dujun Bian, Shixiong Tang

**Affiliations:** ^1^Department of Psychiatry, National Clinical Research Center for Mental Disorders, The Second Xiangya Hospital, Central South University, Changsha, Hunan, China; ^2^Department of Neurology, The Second Xiangya Hospital, Central South University, Changsha, China; ^3^Department of Radiology, Clinical Research Center for Medical Imaging in Hunan Province, The Second Xiangya Hospital, Central South University, Changsha, Hunan, China

**Keywords:** addiction, problematic smartphone use, mobile phone use, fMRI, dynamic functional connectivity

## Abstract

**Introduction:**

This study aimed to investigate the possible associations between problematic smartphone use and brain functions in terms of both static and dynamic functional connectivity patterns.

**Materials and methods:**

Resting-state functional magnetic resonance imaging data were scanned from 53 young healthy adults, all of whom completed the Short Version of the Smartphone Addiction Scale (SAS-SV) to assess their problematic smartphone use severity. Both static and dynamic functional brain network measures were evaluated for each participant. The brain network measures were correlated the SAS-SV scores, and compared between participants with and without a problematic smartphone use after adjusting for sex, age, education, and head motion.

**Results:**

Two participants were excluded because of excessive head motion, and 56.9% (29/51) of the final analyzed participants were found to have a problematic smartphone use (SAS-SV scores ≥ 31 for males and ≥ 33 for females, as proposed in prior research). At the global network level, the SAS-SV score was found to be significantly positively correlated with the global efficiency and local efficiency of static brain networks, and negatively correlated with the temporal variability using the dynamic brain network model. Large-scale subnetwork analyses indicated that a higher SAS-SV score was significantly associated with higher strengths of static functional connectivity within the frontoparietal and cinguloopercular subnetworks, as well as a lower temporal variability of dynamic functional connectivity patterns within the attention subnetwork. However, no significant differences were found when directly comparing between the groups of participants with and without a problematic smartphone use.

**Conclusion:**

Our results suggested that problematic smartphone use is associated with differences in both the static and dynamic brain network organizations in young adults. These findings may help to identify at-risk population for smartphone addiction and guide targeted interventions for further research. Nevertheless, it might be necessary to confirm our findings in a larger sample, and to investigate if a more applicable SAS-SV cutoff point is required for defining problematic smartphone use in young Chinese adults nowadays.

## Introduction

In the past years, the popularity and availability of smartphones have been increasing worldwide, and such a trend is accompanied by increased concerns regarding the potential overuse of smartphones (Horvath et al., 2020; Ratan et al., 2021). Recently, the term “problematic smartphone use” (or also called “problematic mobile phone use” by some researchers) has been introduced, which is defined as excessive use of smartphones with features of craving, dependence, loss of control, and potentially related physical and mental health problems (Long et al., 2016; Harris et al., 2020; Zou et al., 2021). These problems include, for instance, bodily pain (Ng et al., 2020), poor sleep quality (Huang et al., 2020), reduced physical fitness (Wacks and Weinstein, 2021), as well as mental problems such as depressive symptoms (Elhai et al., 2017; Yang X. et al., 2021) and even major depressive disorder (Alageel et al., 2021).

Identifying factors associated with problematic smartphone use can help identify at-risk population and guide targeted interventions for further research (Luk et al., 2018; Roh et al., 2018). Resting-state functional magnetic resonance imaging (rs-fMRI) offers a promising approach for characterizing the intrinsic brain functional organizations (Canario et al., 2021; Lin et al., 2021). Using rs-fMRI, a growing body of neuroimaging studies has suggested that problematic smartphone use is associated with brain dysfunction even in non-clinical samples with no diagnosis of psychiatric disorders (Chun et al., 2018; Paik et al., 2019; Horvath et al., 2020; Ahn et al., 2021; Pyeon et al., 2021; Zou et al., 2022). For example, the severity of problematic smartphone use has been reported to be positively associated with functional connectivity between the parahippocampal gyrus and middle temporal gyrus (Zou et al., 2022), and negatively associated with the fronto-limbic functional connectivity (Pyeon et al., 2021) in general populations. In another study, problematic smartphone use was suggested to be related to enhanced functional connectivity within the salience network, as well as between the salience and default-mode networks (Ahn et al., 2021). Importantly, some of these alterations (e.g., parahippocampal gyrus-middle temporal gyrus functional connectivity) have been found to moderate the relationship between problematic smartphone use and depressive symptoms in adolescents (Zou et al., 2022). Appreciably, these findings have advanced our understanding of the potential neurobiological factors associated with problematic smartphone use, which may guide further research on interventions for this problem.

The currently published rs-fMRI studies on problematic smartphone use, however, are limited in several ways. Firstly, most of these studies were focused on connectivity patterns within predefined regions of interest (ROIs). Although there have been some attempts (Ahn et al., 2021), investigations on how problematic smartphone use would affect the large-scale configurations of brain networks are relatively limited. Especially, it has been suggested that graph-theoretical-based features of the whole-brain network (e.g., global and local efficiency) can provide a powerful and reliable framework for understanding the alterations in brain function (Achard and Bullmore, 2007; Cao et al., 2014; Yang H. et al., 2021), but their possible relationships with problematic smartphone use were seldom reported. Secondly and importantly, while conventional rs-fMRI studies were generally performed under the assumption that connectivity patterns between brain areas are static, recent studies have proved that the brain connectivity patterns are actually dynamically changed over time (Hutchison et al., 2013a,b). The “dynamic functional connectivity (dFC)” was suggested to reflect important information ignored by conventional “static functional connectivity (sFC)” (Park et al., 2018; Zhang W. et al., 2018), and has been widely used in recent rs-fMRI studies in both psychiatric (Sheng et al., 2021; Chen et al., 2022) and non-clinical (Long et al., 2019; Huang D. et al., 2021) populations. Nonetheless, whether problematic smartphone use would affect the brain dFC patterns have been barely investigated to our knowledge, and needs further investigation.

To overcome the above limitations, this study aimed to investigate the possible associations between problematic smartphone use and differences in large-scale brain network organizations by combining both sFC and dFC analyzing methods. We anticipate that the results would provide meaningful information to previous studies focusing on only specific ROIs and/or on only brain sFC patterns, and further improve our understanding of the possible biological factors associated with problematic smartphone use.

## Materials and methods

### Participants and measures

Fifty-three young healthy adults were recruited from the Changsha city area, Hunan Province, China based on the following inclusion criteria: (1) 18∼25 years of age; (2) native Chinese speakers; (3) right-handed; (4) were never diagnosed with any psychiatric diseases; and (5) had no contraindications to rs-fMRI scanning. All participants had signed informed consent, and the study was proved by the Ethics Committee of Second Xiangya Hospital, Changsha, China.

The participants were asked to complete the Short Version of the Smartphone Addiction Scale (SAS-SV) (Kwon et al., 2013) to assess the problematic smartphone use severity. The SAS-SV was a self-reported scale that contains 10 items, each rated from 1 (“strongly disagree”) to 6 (“strongly agree”). Thus, the total score of SAS-SV ranges from 10 to 60, and a higher score indicates a higher level of current problematic smartphone use (Kwon et al., 2013; Luk et al., 2018). The Chinese version of SAS-SV has been proved to be valid (Luk et al., 2018) and was widely applied in Chinese adults (Chen et al., 2017; Guo et al., 2020, 2021; Zhang et al., 2022).

All participants also completed the following scales to estimate their current mental health situations during the past two weeks: (1) the 9-item Patient Health Questionnaire (PHQ-9), a screening instrument for depressive symptoms (Kroenke et al., 2001; Wu et al., 2022); and (2) the seven-item Generalized Anxiety Disorder Scale (GAD-7), a questionnaire to assess anxiety levels (Spitzer et al., 2006; Wu et al., 2022). The Cronbach’s α coefficients of the SAS-SV, PHQ-9, and GAD-7 in this study were 0.869, 0.831, and 0.867 respectively, which suggests a good internal consistency (Cronbach’s α coefficient > 0.7) (Wu et al., 2022).

### Imaging data acquisition and preprocessing

The rs-fMRI data were acquired from each participant using a 3.0 T Siemens scanner with the following key parameters: matrix = 64 × 64, slices = 32, repetition time (TR) = 2,000 ms, echo time (TE) = 30 ms, slice thickness = 5 mm, gap = 0 mm, flip angle = 90°, field of view (FOV) = 240 × 240 mm^2^, and total volumes = 216. T1-weighted images were also acquired for registration with the following key parameters: matrix = 256 × 256, slices = 176, TR = 1,900 ms, TE = 2 ms, slice thickness = 1 mm, gap = 0 mm, and FOV = 256 × 256 mm^2^. After data acquisition, the images of all participants were preprocessed using the DPARSF software^1^ (Chao-Gan and Yu-Feng, 2010; Yan et al., 2016) with the standard pipeline. Briefly, the pipeline includes removing the first 10 time points, slice timing, motion realignment, spatial normalization, temporal filtering (0.01–0.10 Hz), and nuisance regression (including the white matter and cerebrospinal fluid signals) (Yan et al., 2019; Long et al., 2020a). The following procedures were performed to ensure data quality: (1) all preprocessed images were manually checked by trained researchers to rule out overt artifacts or poor registration; (2) data were excluded from the analyses when excessive head motion occurred during scanning, as defined by mean framewise-displacement (FD) > 0.2 mm (Huang X. et al., 2021); (3) the mean FD values were further used as a controlling variable in all the following analyses. More details about the data acquisition parameters and preprocessing steps can be found in a previously published work (Huang D. et al., 2021).

### Static and dynamic brain network constructions

**FIGURE 1:**
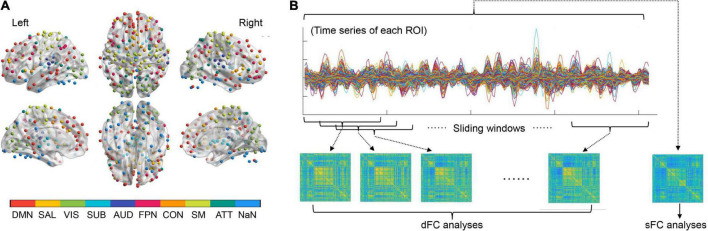
**(A)** The regions of interests (ROIs) used to define the brain nodes and their subnetwork assignments. **(B)** A summary of procedures for static functional connectivity (sFC) and dynamic functional connectivity (dFC) analyses (see details in the Methods section). ATT, attention subnetwork; AUD, auditory subnetwork; CON, cinguloopercular subnetwork; DMN, default-mode subnetwork; FPN, frontoparietal subnetwork; NaN, unassigned; SAL, salience subnetwork; SM, sensorimotor subnetwork; SUB, subcortical subnetwork; VIS, visual subnetwork.

The Power functional atlas (Power et al., 2011), which includes a total of 264 ROIs distributed across the brain (see Figure 1A), was used to define the nodes in brain networks for each participant. We chose the Power atlas here since it was widely used and validated in both sFC and dFC studies (Cao et al., 2014; Tan et al., 2020; Long et al., 2021). The mean time series were firstly extracted from each of the 264 nodes (ROIs) by averaging rs-fMRI signals within each node. The sFC strength for any pair of two nodes was computed as the Fisher’s r-to-z transformed Pearson’s correlation coefficients of the extracted time series, yielding a 264*264 sFC matrix which represents the static brain network organization (Figure 1B).

To construct dynamic brain networks, the extracted time series were further segmented into a number of continuous time windows using a common sliding-window approach (Long et al., 2020a; Zhao et al., 2021). A window width of 50 TRs (100 s) and a step length of 3 TRs (6 s) were used based on previous recommendations (Sun et al., 2019; Long et al., 2020a; Tang et al., 2022), resulting in a total of 53 time windows. Similar to the sFC matrixes, a 264*264 dFC matrix was then generated for each time window based on the Fisher’s r-to-z transformed connection strengths between nodes. These dFC matrices are time-ordered, and thus formed a dynamic brain network *G* = (*G*_*t*_)_*t* = 1, 2, 3, …, 53_, in which the *t*th matrix (*G*_*t*_) represents the “snapshot” of brain dFC patterns within the *t*th time window (Sun et al., 2019; Huang D. et al., 2021; Figure 1B).

### Global and nodal brain network metrics

Several common global and nodal network metrics were calculated for both the static and dynamic (weighted, undirected) brain networks for each participant. Static network metrics included the global efficiency (*E*_*glob*_) and local efficiency (*E*_*loc*_) at the global level, as well as the nodal degree of each node. The *E*_*glob*_ and *E*_*loc*_ are two of the most intuitive and widely-used metrics to measure the information transfer efficiency of a static brain network (Tan et al., 2020; Yang H. et al., 2021; Liu D. et al., 2022). The nodal degree is a basic measure of the overall connectivity of a node to the rest of the brain (Li T. et al., 2021; Yang H. et al., 2021). The *E*_*glob*_ and *E*_*loc*_ were calculated in a range of density levels from 0.10 to 0.34 with an interval of 0.01, to avoid possible bias caused by a single density level (Achard and Bullmore, 2007; Lv et al., 2021; Yang H. et al., 2021). This range was chosen because it guaranteed that the network metrics were estimable and there were not too many spurious edges (Achard and Bullmore, 2007; Zhang et al., 2011). For each metric, the area under the curve (AUC) across such a density range (0.10–0.34) was calculated and fed into statistical analyses (Zhang et al., 2011; Yang H. et al., 2021). Referring to the previous work, the characteristic path length (*L*_*p*_) and clustering coefficient (*C*_*p*_) were also calculated for the latter validation analyses (Yang H. et al., 2021). The above static brain network metrics were calculated using the Brain Connectivity Toolbox (Rubinov and Sporns, 2010).

The examined dynamic network metrics included the *temporal variability* for the entire brain network and *nodal temporal variability* of each node (Zhang et al., 2016; Dong et al., 2019; Long et al., 2020b; Sun et al., 2022). These two metrics quantify the temporal stability of brain dFC patterns at the global and nodal levels, respectively; higher values of temporal variability indicate more fluctuations of the dFC patterns (less stable dFCs) over time. More details about the calculations of these two metrics can be found in previous publications (Zhang et al., 2016; Dong et al., 2019; Long et al., 2020b; Sun et al., 2022).

### Large-scale subnetwork analyses

Besides the global and nodal network metrics, large-scale subnetwork analyses were also performed on both the sFC and dFC architectures strictly following the procedures in previous publications (Dong et al., 2019; Long et al., 2020b; Li L. et al., 2021; Sun et al., 2022). According to prior work (Cole et al., 2013; Mohr et al., 2016; Long et al., 2019, 2021), all ROIs in the Power atlas were firstly assigned into nine large-scale subnetworks including the default-mode, salience, visual, subcortical, auditory, frontoparietal, cinguloopercular, sensorimotor and attention subnetworks (Figure 1A). The strengths of within-and between-subnetwork sFC were calculated by averaging the z-transformed sFC values across all involved connections within a specific subnetwork, or between a specific pair of subnetworks (Li L. et al., 2021). Similarly, the temporal variabilities of within- and between-subnetwork dFC were also obtained by calculating the average variabilities of dFC across all involved connections (Dong et al., 2019; Long et al., 2020b; Sun et al., 2022). This resulted in nine within-subnetwork sFC/dFC measures and 36 between-subnetwork sFC/dFC measures.

### Statistics

The possible associations between problematic smartphone use and all the sFC/dFC measures were investigated from two perspectives. Firstly, relationships between all brain network measures and the SAS-SV score were assessed using the partial Pearson correlations adjusted for age, sex, years of education, and head motion (mean FD value). False discovery rate (FDR) corrections were applied to correct for multiple correlation tests (e.g., across the three global metrics, the 264 nodes, the nine within-subnetwork and 36 between-subnetwork measures). Significance was set at FDR-corrected *p* < 0.05. The results were visualized partly using the BrainNet Viewer (Xia et al., 2013).

Secondly, all brain network measures were compared between the groups of participants with and without a problematic smartphone use, as defined by the commonly-used SAS-SV cutoff points proposed in prior research (SAS-SV scores ≥ 31 for males and ≥ 33 for females) (Kwon et al., 2013; Luk et al., 2018; Saadeh et al., 2021; Liu H. et al., 2022). All brain network measures were compared between the two groups using the analysis of covariance (ANCOVA) covarying for age, sex, years of education, and head motion. Similarly, FDR corrections were applied to correct for multiple comparisons, and significance was set at FDR-corrected *p* < 0.05.

### Validation analyses

Several follow-up analyses were performed to validate the results. Firstly, the associations between the SAS-SV score and *L*_*p*_/*C*_*p*_, which have equivalent meanings to the *E*_*glob*_ and *E*_*loc*_ (Yang H. et al., 2021), were estimated using the same methods. Secondly, since the optimal window width and step length for the sliding-windows method are still being debated (Leonardi and Van De Ville, 2015; Zhang C. et al., 2018), the analyses on all dFC measures were repeatedly with a set of different window and step lengths for the sliding windows [window/step = (80, 100, 120)/(4, 6, 8) s] to see if the results were affected by such analyzing strategies.

### Exploratory analyses

**FIGURE 2:**
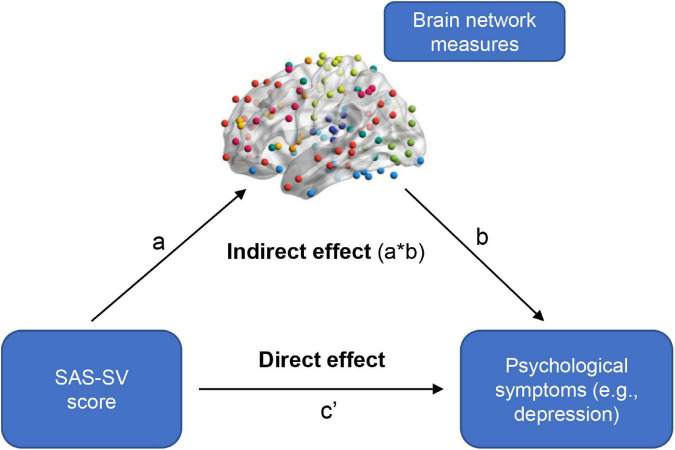
A summary of how to test the mediation effects of brain network measures in the relationship between problematic smartphone use severity and psychological symptoms. A significant mediation occurred when the 95% confidence interval for the indirect effect did not include zero.

In the present study, we performed two-step exploratory analyses to see if those problematic smartphone use-related differences in sFC/dFC would have mediation effects in the relationship between problematic smartphone use and psychological symptoms. Firstly, the linear regression analyses (controlling for age, sex, and education) were used to determine whether an association existed between the SAS-SV score and the GAD-7/PHQ-9 score. Secondly, when significant associations existed (*p* < 0.05), the analyses of mediation effects were further conducted using the PROCESS software (Hayes, 2012) on the sFC/dFC measures. Model 4 in the PROCESS software was used with 5,000 bootstrapping resamples; a significant mediation occurred when the 95% confidence interval (CI) for the indirect effect did not include zero (Figure 2; Rhudy et al., 2020; Li J. et al., 2021; Wu et al., 2021).

Additionally, since no significant results were found in group comparisons between the participants with and without a problematic smartphone use (see later in Section “Group comparisons”) based on the SAS-SV cutoff points proposed in prior research (≥ 31 for males and ≥ 33 for females), we explored whether the results would change when using a different cutoff. Here, referring to some published studies (Mullins et al., 2007; Asarnow et al., 2019; Quintero Garzón et al., 2021), we used a cutoff score estimated based on one standard deviation above the mean of SAS-SV score in the surveyed sample; this resulted a new cutoff score of ≥ 43 for males and ≥ 42 for females. Group comparisons were repeated based on such new cutoff.

## Results

### Sample characteristics

During data preprocessing, two participants were excluded because of excessive head motion. Thus, the final analyzed sample consisted of 51 subjects and their demographic and clinical characteristics are presented in Table 1.

**TABLE 1:** Characteristics of the final analyzed sample (*n* = 51).

	Mean ± Standard deviation
Age	21.51 ± 1.55
Sex (males/females)	16/35
Year of education	15.65 ± 1.93
SAS-SV score	32.78 ± 9.21
SAS-SV score (in males)	34.69 ± 7.87
SAS-SV score (in females)	31.91 ± 9.74
GAD-7 score	3.12 ± 3.20
PHQ-9 score	4.18 ± 3.66

### Correlation analyses

**FIGURE 3:**
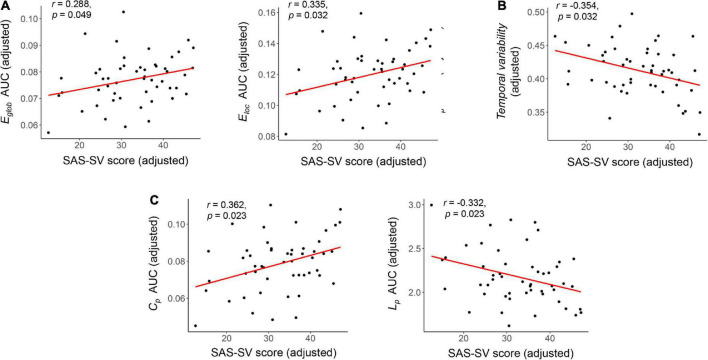
Results of the partial correlations between the short version of the smartphone addiction scale (SAS-SC) score and each global brain network metric. **(A)** Results on the *E*_*glob*_ and *E*_*loc*_ of static brain networks. **(B)** Results on the temporal variability of dynamic brain networks. **(C)** Results on the *C*_*p*_ and *L*_*p*_ of static brain networks (as validation analyses). The partial Pearson correlation coefficients **(*r*)** and corrected *p* values are presented.

At the global level, significant correlations were found between the SAS-SV score and the *E*_*glob*_ of static brain networks (*r* = 0.288, corrected *p* = 0.049), as well as between the SAS-SV score and the *E*_*loc*_ of static brain networks (*r* = 0.335, corrected *p* = 0.032) (Figure 3A). Furthermore, a significant negative correlation was found between the SAS-SV score and the temporal variability of dynamic brain networks (*r* = −0.354, corrected *p* = 0.032) (Figure 3B). At the nodal level, however, no significant correlations were found for any metric (all corrected *p* > 0.05).

**FIGURE 4:**
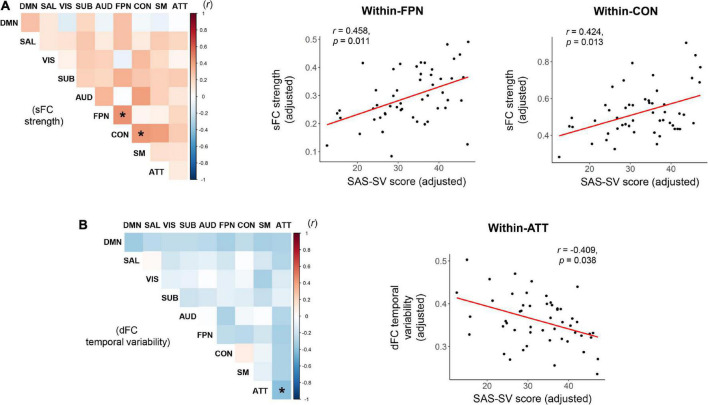
Results of partial correlations between the short version of the smartphone addiction scale (SAS-SC) score and the within-or between-subnetwork static functional connectivity (sFC) strength **(A)** and dynamic functional connectivity (dFC) temporal variability **(B)**. The scatter plots for the significant correlations were also presented on the right side. ATT, attention subnetwork; AUD, auditory subnetwork; CON, cinguloopercular subnetwork; DMN, default-mode subnetwork; FPN, frontoparietal subnetwork; SAL, salience subnetwork; SM, sensorimotor subnetwork; SUB, subcortical subnetwork; VIS, visual subnetwork. *Indicates a significant correlation with corrected *p* < 0.05.

As shown in the Figure 4, significant positive correlations were found between the SAS-SC score and sFC strength within the frontoparietal subnetwork (*r* = 0.458, corrected *p* = 0.011), as well as between the SAS-SC score and sFC strength within the cinguloopercular subnetwork (*r* = 0.424, corrected *p* = 0.013); moreover, a significant negative correlation was found between the SAS-SC score and dFC temporal variability within the attention subnetwork (*r* = −0.409, corrected *p* = 0.038). No significant results were found on the between-subnetwork sFC/dFC measures (all corrected-*p* > 0.05).

### Group comparisons

**FIGURE 5:**
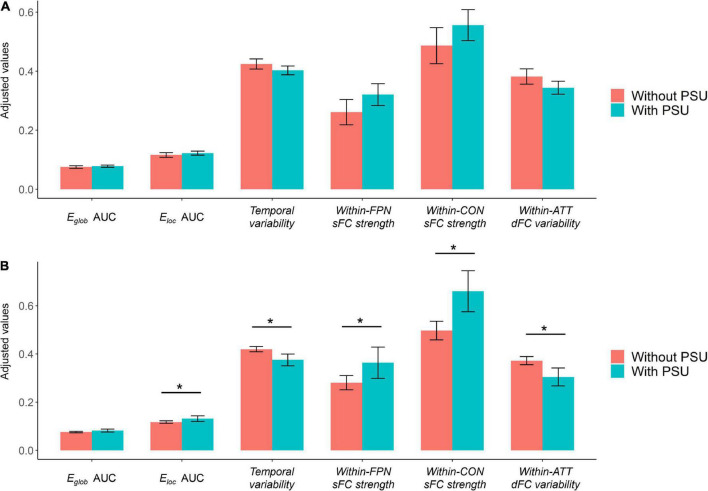
**(A)** Results of group comparisons when defining problematic smartphone use with the cutoff proposed in prior research [short version of the smartphone addiction scale (SAS-SV) score ≥ 31 for males and ≥ 33 for females]. **(B)** Results of exploratory group comparisons when defining problematic smartphone use with a new cutoff (SAS-SV score ≥ 43 for males and ≥ 42 for females, as estimated by one standard deviation above the mean scores). The error bars represent the 95% confidence intervals, and the “*” indicates a significant difference with corrected *p* < 0.05. ATT, attention subnetwork; CON, cinguloopercular subnetwork; PSU, problematic smartphone use.

Based on the cutoff of a SAS-SV score ≥ 31 for males and ≥ 33 for females, 56.9% (29/51) of the participants were found to have a problematic smartphone use. However, no significant group differences were found on any brain network measure between the participants with and without a problematic smartphone use (all corrected *p* > 0.05), even for those measures showing significant correlations with the SAS-SV score (Figure 5A).

### Validation analyses

Significant correlations were found between the SAS-SV score and *L*_*p*_ (*r* = 0.363, corrected *p* = 0.023), as well as between the SAS-SV score and *C*_*p*_ (*r* = −0.332, corrected *p* = 0.023) (Figure 3C), which thus partly validate the findings on *E*_*glob*_ and *E*_*loc*_.

The relationships between the SAS-SV score and dFC measures remained significant when repeating the analyses with a set of different window and step lengths (see Supplementary Tables 1, 2). Therefore, the results were unlikely to be largely affected by the analyzing parameters.

### Exploratory analyses

The linear regression analyses revealed a significant positive relationship between the SAS-SV score and the PHQ-9 score (β = 0.154, *t* = 2.787, *p* = 0.008), suggesting that problematic smartphone use is associated with a higher level of depressive symptoms. However, no significant mediation effects were observed for any sFC/dFC measure in the relationship between problematic smartphone use and depressive symptoms (no significant indirect effects were observed, as shown in Table 2).

**TABLE 2:** Results of the mediation effect analyses on each brain network measures in the relationship between the short version of the smartphone addiction scale (SAS-SV) score and patient health questionnaire (PHQ-9) score.

Brain network measures	Direct effects (with 95% confidence intervals)	Indirect effects (with 95% confidence intervals)
**Global network metrics**		
Global efficiency	0.140 (0.022, 0.259)*	0.015 (−0.013, 0.065)
Local efficiency	0.141 (0.020, 0.261)*	0.015 (−0.021, 0.068)
Temporal variability	0.152 (0.030, 0.275)*	0.003 (−0.040, 0.059)
Characteristic path length	0.142 (0.022, 0.263)*	0.013 (−0.021, 0.066)
Clustering coefficient	0.141 (0.019, 0.263)*	0.014 (−0.025, 0.065)
**Subnetwork-level measures**		
Within-FPN sFC strength	0.138 (0.010, 0.267)*	0.017 (−0.045, 0.087)
Within-CON sFC strength	0.155 (0.029, 0.282)*	0.000 (−0.060, 0.058)
Within-ATT dFC temporal variability	0.150 (0.025, 0.275)*	0.006 (−0.043, 0.054)

The “*” indicates a significant direct or indirect effect (with a 95% confidence interval not including zero). ATT, attention subnetwork; CON, cinguloopercular subnetwork; FPN, frontoparietal subnetwork.

When defining problematic smartphone use with a new cutoff (SAS-SV score ≥ 43 for males and ≥ 42 for females), 17.6% (9/51) of the participants were considered to have a problematic smartphone use. When using such new cutoff points, significant group differences were found between the participants with and without a problematic smartphone use on most brain network measures which showed significant correlations with the SAS-SV score (corrected *p* < 0.05, Figure 5B).

## Discussion

In this study, we investigated the possible associations between problematic smartphone use and brain functions in young healthy adults combining both the sFC and dFC analyzing methods. Overall, our results suggested that the severity of smartphone use is associated with significant differences in both the static and dynamic brain network organizations.

For static brain network properties, our results suggested that higher smartphone use severity is significantly associated with a higher *E*_*glob*_ as well as a higher *E*_*loc*_ at the global level (Figure 3A). Such results were further validated by significant results on the *C*_*p*_ and *L*_*p*_, which were known to have equivalent meanings to the *E*_*glob*_ and *E*_*loc*_ (Yang H. et al., 2021; Figure 3C). While the neuroimaging studies on problematic smartphone use are growing (Ahn et al., 2021; Pyeon et al., 2021; Zou et al., 2022), the possible effects of problematic smartphone use on these graph-theoretical-based brain network features are still seldom reported. Nevertheless, similar alterations in the brain networks (increased *E*_*glob*_ and/or *E*_*loc*_) have been associated with some common psychiatric diseases such as posttraumatic stress disorder (Lei et al., 2015), as well as multiple substance/non-substance addictions such as the nicotine dependence (Lin et al., 2015) and Internet gaming addiction (Park et al., 2017). Our results may thus provide preliminary evidence that higher smartphone use severity could be related to changing trends in topological functional brain organizations, which is similar to changes in patients with these disorders. These findings may help to identify at-risk population for smartphone addiction, and guide targeted interventions for further research.

Using the dynamic network model, our results suggested that problematic smartphone use is associated with a lower temporal variability (Figure 3B), which indicates a decreased dynamism of brain networks (Long et al., 2020b). Previous studies have proved that there are unignorable dynamic fluctuations in the human brain’s functional organizations (Hutchison et al., 2013a,b), which is closely related to the cognitive (Patil et al., 2021) and emotional (Tobia et al., 2017) processes. Meanwhile, both excessively increased (Long et al., 2020b; Sun et al., 2022) and decreased (Jin et al., 2017; Luo L. et al., 2021; Luo Z. et al., 2021) dynamisms were thought to be reflective of abnormal brain functions. Specially, a decreased dynamism may indicate a disturbance in the information processing across brain regions (Luo Z. et al., 2021). Here, our results therefore provide one of the first evidence that problematic smartphone use may decrease the functional brain network dynamism.

At the subnetwork level, it was found that a higher smartphone use severity is associated with increased sFC strengths within the frontoparietal and cinguloopercular subnetworks (Figure 4A), as well as decreased dFC temporal variability within the attention subnetwork (Figure 4B). The frontoparietal and cinguloopercular subnetworks are known to be implicated in higher-level cognitive functions (Wallis et al., 2015; Schmidt et al., 2016). The attention subnetwork is thought to be responsible for the top-down attentional process, whose abnormality is associated with attention deficits (Vossel et al., 2014; Baldassarre et al., 2016). Therefore, it may be hypothesized that these brain subsystems are prominently disrupted by problematic smartphone use, which may be partially related to the smartphone use-caused cognitive impairments (Wacks and Weinstein, 2021) and attention deficits (Choi et al., 2021). However, this assumption remains speculative and needs to be tested in further studies, since no cognitive or attentional tests were performed in this study. Additionally, it is noteworthy that in the current study, the sFC and dFC analyses suggested significant smartphone use-associated effects in difference brain subnetworks, indicating that they may reflect different aspects of brain function. This may partly support the opinion that dFC can capture important information ignored by conventional static methodology (Hutchison et al., 2013a), and further highlight the value of integrating the sFC and dFC analyses in research on problematic smartphone use.

While significant correlations were found between the brain network metrics and SAS-SV score, no significant differences were obtained when directly comparing between the groups of participants with and without a problematic smartphone use (Figure 5A). One possible reason is that our sample size is relatively small, which may limit the statistical power of this research; a larger sample might be needed to detect the between-group differences. We also note that based on the commonly-used SAS-SV cutoff points (≥ 31 for males and ≥ 33 for females), a considerable proportion (56.9%) of participants were found to have a problematic smartphone use. However, such a proportion is much higher than most previous research [e.g., 29.8% in Mainland China (Chen et al., 2017), 24.8% in South Korea (Kwon et al., 2013), and 38.5% in Hong Kong populations (Luk et al., 2018)]. Here, we thus propose that such a cutoff may be not optimal for the current sample of young Chinese adults. The previous SAS-SV threshold points proposed by the scale developers (Kwon et al., 2013) may lead to an over-estimated prevalence of problematic smartphone use nowadays, considering that the use of smartphone has been largely increased in recent years and is being frequently engaged with everyday life and work. In fact, such an opinion has also been expressed by other researchers (Saadeh et al., 2021), and may be partly supported by the results of our exploratory analyses using more strict cutoff points (Figure 5B). Therefore, further studies may be warranted to investigate if a more applicable SAS-SV cutoff point is required for defining problematic smartphone use in young Chinese nowadays.

Previous studies have reported that alterations in brain structures may act as a moderator of the relationship between problematic smartphone use and depressive symptoms in young adults (Zou et al., 2021). In the current study, on the contrary, no similar mediation effects were found on any sFC/dFC measure (Table 2). Nonetheless, it is noteworthy that the sample size is relatively low; moreover, only healthy participants were included whose depressive levels were relatively low. Further studies may be warranted to detect possible mediation effects in a larger sample and in clinical populations.

Some other limitations of this study should be noted. First, because of the nature of cross-sectional research, we are unable to determine the causality relationship between problematic smartphone use and brain dysfunctions. Second, as the SAS-SV is a self-reported scale, the results could be biased by potential over-or under-reports. Third, while only the sFC/dFC patterns during rest were analyzed, further studies conducted under specific tasks (Choi et al., 2021) may further improve our knowledge. Fourth, in this study, we chose the sliding-window approach to analyze dFC rather than other approaches such as the temporal independent component analysis (tICA), considering that the tICA requires a large number of scanning time points (Li et al., 2020) and the sliding-window approach might be more suitable for the current dataset. Nevertheless, other approaches such as the tICA may provide further important information and can be investigated in the future studies.

In conclusion, this study showed that problematic smartphone use is associated with differences in brain functions in young healthy adults, as characterized by differences in both static and dynamic brain network organizations. These findings may help to improve our understanding of the biological associates of problematic smartphone use. However, further studies may be warranted to confirm our findings in a larger sample, and to investigate if a more applicable SAS-SV cutoff point is required for defining problematic smartphone use in young Chinese nowadays.

## Data availability statement

The raw data supporting the conclusions of this article will be made available by the authors, without undue reservation.

## Ethics statement

The studies involving human participants were reviewed and approved by The Ethics Committee of Second Xiangya Hospital. The patients/participants provided their written informed consent to participate in this study.

## Author contributions

DL, YL, and ST designed the study and carried out the analysis. DL, YL, DB, and ST contributed to the data collection. DL, XL, and YL wrote the first draft of manuscript. ZX, ZW, ZL, and ST contributed to the final manuscript. All authors have read and agreed to the published version of the manuscript.

## Footnotes

1
http://rfmri.org/DPARSF
