# Supplementary material for: Problematic smartphone use is associated with differences in static and dynamic brain functional connectivity in young adults
Source: Front Neurosci. 2022 Oct 21;16:1010488. doi: 10.3389/fnins.2022.1010488 (PMC9635624; doi:10.3389/fnins.2022.1010488)
Supplement: Supplementary file 1 [file Data_Sheet_1.docx]

**Supplementary Table 1** Results of partial correlations between the SAS-SC score and temporal variability of the global brain network when dynamic brain networks were constructed with different window widths/step lengths.

| Window width | Step length |  |  |
| --- | --- | --- | --- |
|  | 4 s | 6 s | 8 s |
| 80 s | *r* = -0.362,  *p* = 0.012 | *r* = -0.363,  *p* = 0.012 | *r* = -0.363,  *p* = 0.012 |
| 100 s | *r* = -0.354,  *p* = 0.015 | */* | *r* = -0.353,  *p* = 0.015 |
| 120 s | *r* = -0.361,  *p* = 0.013 | *r* = -0.360,  *p* = 0.013 | *r* = -0.359,  *p* = 0.013 |

**Supplementary Table 2** Results of partial correlations between the SAS-SC score and temporal variability of dFC within the attention subnetwork when dynamic brain networks were constructed with different window widths/step lengths. The presented *p* values were FDR-corrected.

| Window width | Step length |  |  |
| --- | --- | --- | --- |
|  | 4 s | 6 s | 8 s |
| 80 s | *r* = -0.433,  *p* = 0.018 | *r* = -0.431,  *p* = 0.027 | *r* = -0.433,  *p* = 0.037 |
| 100 s | *r* = -0.410,  *p* = 0.036 | */* | *r* = -0.409,  *p* = 0.036 |
| 120 s | *r* = -0.429,  *p* = 0.027 | *r* = -0.429,  *p* = 0.027 | *r* = -0.429,  *p* = 0.027 |
